# Mitochondrial dysfunction as the missing link between circadian syndrome and dementia

**DOI:** 10.1016/j.tjpad.2025.100125

**Published:** 2025-03-06

**Authors:** Yu-Hsiang Lin, Kuo-Jen Lin, Po-Ting Lin

**Affiliations:** a Department of Urology, Chang Gung Memorial Hospital-Linkou, Taiwan; b School of Medicine, Chang Gung University, Taoyuan 333, Taiwan

Dear editor,

We read with great interest the study by Yu et al. [1] on the interaction between circadian syndrome (CircS) and genetic susceptibility in dementia risk. The authors provided compelling epidemiological evidence supporting the association between CircS severity and increased dementia risk. However, a crucial mechanistic pathway remains underexplored—the role of mitochondrial dysfunction as a central mediator linking CircS and neurodegenerative diseases.

The suprachiasmatic nucleus (SCN), the central pacemaker of circadian rhythms, modulates multiple endocrine axes, including the hypothalamic-pituitary-gonadal (HPG), hypothalamic-pituitary-adrenal (HPA), and hypothalamic-pituitary-thyroid (HPT) axes. Disruptions in these hormonal pathways—such as imbalances in testosterone, estrogen, glucocorticoids, and thyroid hormones—can induce mitochondrial dysfunction, a well-documented contributor to both metabolic diseases and neurodegenerative disorders [[2], [3], [4]].

Mitochondria are highly sensitive to hormonal fluctuations. Recent evidence suggests that testosterone, thyroid hormones, and glucocorticoids directly regulate mitochondrial oxidative phosphorylation (OXPHOS) and bioenergetics [5]. Al-Suhaimi et al. [5] highlighted that hormonal imbalances lead to mitochondrial stress, triggering metabolic and neurodegenerative pathways, which may explain the observed correlation between CircS severity and increased dementia risk.

Furthermore, aged neurons exhibit impaired excitation-transcription coupling, reducing mitochondrial oxidative phosphorylation efficiency and ATP production. This failure to register energy demands leads to increased oxidative stress and accelerates neurodegeneration [2]. Given that neurons are highly energy-dependent, mitochondrial dysfunction creates a metabolic bottleneck that facilitates cognitive decline and dementia. This provides a biological basis for the epidemiological association between CircS and dementia risk observed in Yu et al. [1].

Emerging evidence suggests that metabolic diseases and neurodegenerative disorders share common pathological mechanisms, including mitochondrial dysfunction, chronic inflammation, and oxidative stress. For instance, insulin resistance and metabolic syndrome have been linked to an increased risk of cognitive decline, while neurodegeneration can further disrupt metabolic homeostasis. This bidirectional interaction underscores the need for integrated therapeutic strategies targeting both conditions.

Given these insights, we propose that mitochondrial dysfunction should be considered a crucial mechanistic link underlying the association between CircS and dementia. Future research should explore whether targeting mitochondrial health—through circadian rhythm modulation, testosterone restoration, or metabolic interventions such as time-restricted feeding or melatonin supplementation—could mitigate the neurodegenerative impact of CircS [3].

Fig. 1 illustrates this proposed mechanistic link, demonstrating how SCN dysregulation disrupts endocrine axes, contributing to mitochondrial dysfunction and subsequently increasing the risk of metabolic and neurodegenerative diseases.

**Fig. 1:**
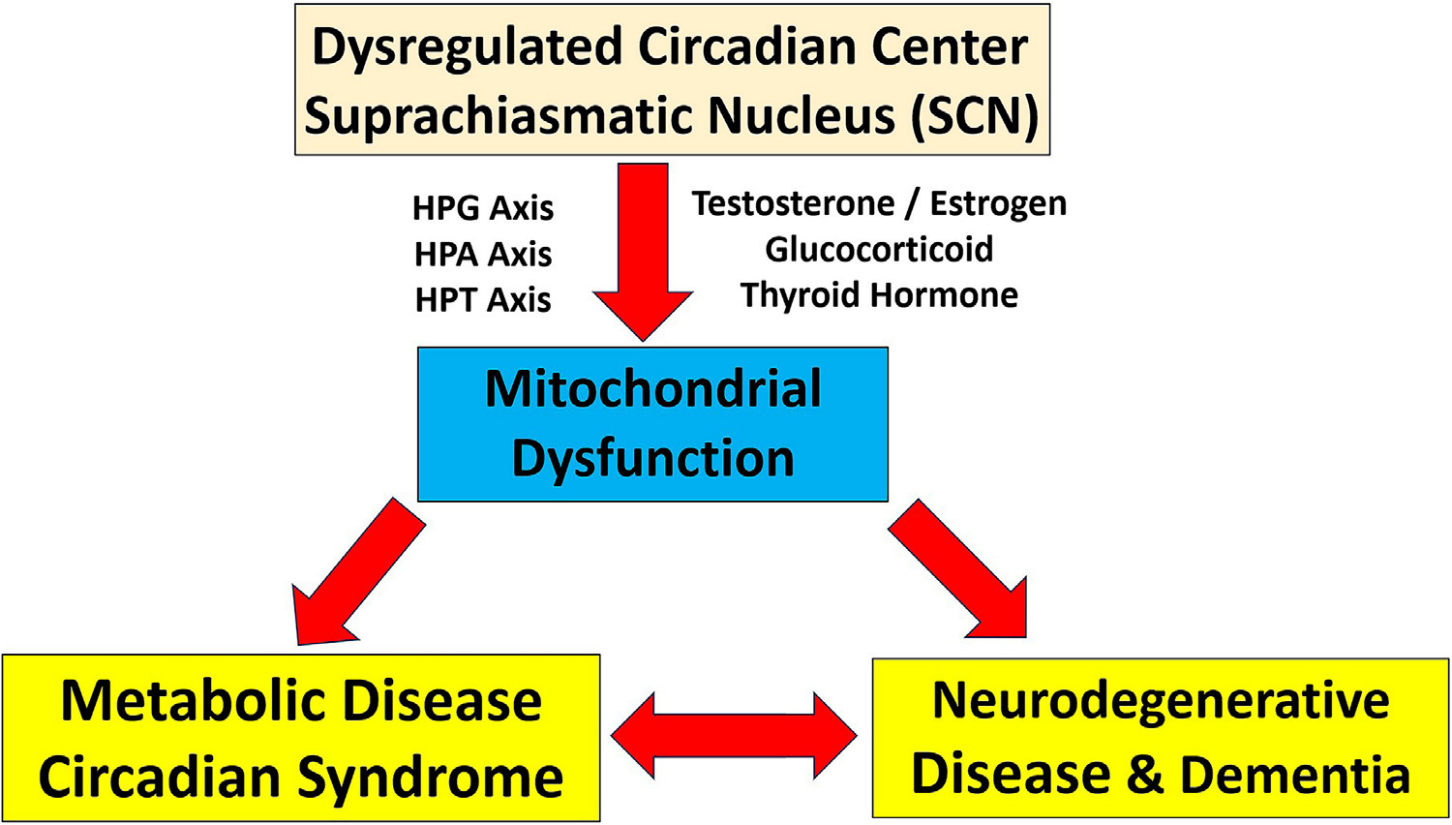
**Mitochondrial Dysfunction as the Missing Link between Circadian Syndrome and Dementia**. Proposed mechanistic link between circadian syndrome (CircS) and dementia via mitochondrial dysfunction. Dysregulation of the suprachiasmatic nucleus (SCN) disrupts endocrine axes, including the hypothalamic-pituitary-gonadal (HPG), hypothalamic-pituitary-adrenal (HPA), and hypothalamic-pituitary-thyroid (HPT) axes, leading to hormonal imbalances in testosterone, estrogen, glucocorticoids, and thyroid hormones. These endocrine disruptions impair mitochondrial function, contributing to metabolic diseases and neurodegenerative disorders. Additionally, a bidirectional relationship exists between metabolic diseases (e.g., Circadian Syndrome) and neurodegenerative diseases (e.g., dementia), highlighting their potential mutual influence.

## Ethical statement

Not applicable.

## Funding/support and role of sponsor

Nil.

## Data sharing statement

This Letter to the Editor is based on previously published literature and does not involve any new data collection by the authors. All analyses and discussions are derived from publicly available sources cited within the manuscript. Therefore, no datasets were generated or analyzed during this study.

## CRediT authorship contribution statement

**Yu-Hsiang Lin:** Conceptualization, Writing – original draft, Writing – review & editing. **Kuo-Jen Lin:** Conceptualization. **Po-Ting Lin:** Conceptualization.

## Declaration of competing interest

The authors declare the following financial interests/personal relationships which may be considered as potential competing interests:

Reports a relationship with that includes:. Has patent pending to. If there are other authors, they declare that they have no known competing financial interests or personal relationships that could have appeared to influence the work reported in this paper.
